# Deciphering Parameter Sensitivity in the BvgAS Signal Transduction

**DOI:** 10.1371/journal.pone.0147281

**Published:** 2016-01-26

**Authors:** Tarunendu Mapder, Srijeeta Talukder, Sudip Chattopadhyay, Suman K. Banik

**Affiliations:** 1 Department of Chemistry, Indian Institute of Engineering Science and Technology, Shibpur, Howrah 711103, India; 2 Department of Chemistry, University of Calcutta, 92 A P C Road, Kolkata 700 009, India; 3 Department of Chemistry, Bose Institute, 93/1 A P C Road, Kolkata 700 009, India; King’s College London, UNITED KINGDOM

## Abstract

To understand the switching of different phenotypic phases of *Bordetella pertussis*, we propose an optimized mathematical framework for signal transduction through BvgAS two-component system. The response of the network output to the sensory input has been demonstrated in steady state. An analysis in terms of local sensitivity amplification characterizes the nature of the molecular switch. The sensitivity analysis of the model parameters within the framework of various correlation coefficients helps to decipher the contribution of the modular structure in signal propagation. Once classified, the model parameters are tuned to generate the behavior of some novel strains using simulated annealing, a stochastic optimization technique.

## Introduction

Living systems sustain in a diverse and dynamically changing niche that they have to cope with to survive. As a result, every organism adopts specialized communication machinery that helps in responding to alteration of the immediate environment. Any rapid or slow change in the surroundings is taken care of through intracellular signal transduction pathways and a number of genetic switches [1]. The signal transduction pathways comprise of some specialized motifs to carry out the process of intracellular communication. Two-component system is one such signaling motif prevalently found in bacteria [2–6]. A typical bacterial two-component system comprises of a trans-membrane sensor protein along with a cognate cytoplasmic response regulator protein. Any change in the immediate surroundings is sensed by the sensor protein, which then communicates the information downstream to its cognate partner through the mechanism of phosphorelay. The response regulator then regulates one or several downstream genes in response to the change in the environment.

The diversity of extracellular environment is very high for the microorganisms that invade into the host as pathogens and proliferate. They have to deal with both the environments: inside and outside the host. The microorganism of the current study, *Bordetella pertussis*—a gram-negative human pathogen, shows dormancy in the atmospheric environment (∼25°C) and becomes virulent inside host (∼37°C) [7–9]. Like many other prokaryotes, *B. pertussis* adopts the environmental distortion through a prominent two-component signal transduction cascade, the BvgAS. The key components of the BvgAS two-component system are BvgS and BvgA, the sensor and the response regulator protein, respectively. The expression of several virulent factors, like toxins and adhesins, are mediated by the BvgAS two-component system. The membrane bound sensor kinase, BvgS, encounters temperature dependent activation and down-modulation only through the presence of modulators like MgSO_4_, nicotinic acid or reduced temperature. The BvgAS sensory transduction system shows three different phases: Bvg^+^, Bvg^−^ and Bvg^i^ through gene regulation in response to stimuli [7–9]. The regime of the intermediate (Bvg^i^) phase is narrow than the repressed (Bvg^−^) phase or activated (Bvg^+^) phase due to the sharp switch of the BvgA population. Each of the phases has a unique pattern of gene regulation. At Bvg^+^ phase, the virulence-activated genes (*vags*) *fhaB*, *ptxA* and *bvgAS* itself show maximum expression. In contrast, at Bvg^−^ phase, the virulence-repressed genes (*vrgs*) *flaA*, *frlAB* are expressed at the maximum level, but no *vags* show good expression. The Bvg^i^ phase is characterized by the maximal expression of *bvgAS*, *bipA* and *fhaB*, and nominal expression of *vrgs* and *ptxA*. In the present study, we focus on *bipA* and *fhaB* along with *bvgAS* to observe the temperature mediated switch from dormant to virulent phase.

Depending on the transcription factor binding affinity, the variety of the Bvg-regulated genes are classified into four classes [7–9]. Class 1 genes include *cyaA* and *ptxA*, containing low-affinity binding sites and are activated at high level of phosphorylated BvgA. Class 2 gene *fhaB* possesses high-affinity BvgA binding site and produces transcripts at a very small level of BvgA-P. The unique class 3 gene *bipA* starts transcription at a moderately low level of BvgA-P bound at the low-affinity binding site, but get repressed at a high level of BvgA-P. *frlAB* belongs to class 4 gene and shows repression at high BvgA-P. The class 3 and class 4 gene expression are not observed in *B. pertussis* through temperature elevation. Only class 3 gene are expressed in *B. pertussis* for intermediate level of MgSO_4_. Thus, at a low level of BvgA, *B. pertiussis* expresses class 1, 2 and 3 minimally but class 4 genes maximally. Class 2 and 3 genes show a high level of transcription in contrast with the low level of class 1 and 4 expression at the moderate level of BvgA. Finally, at a high level of BvgA, class 1 and 2 genes show maximum and class 4 genes minimum level of expression with low level of class 3 gene expression.

The well known BvgAS motif is studied here in accord with the network parameter sensitivity. The functionality of the BvgAS signal transduction motif and its effect on the downstream gene regulation is well reported through experimental work [7–9], thus providing a scope to analyze the network from sensitivity analysis and optimization point of view. In this connection it is important to mention that several theoretical approaches have been undertaken to explore the signal transduction mechanism in typical bacterial two-component system [10–21]. However, few theoretical formalism have been developed to address the underlying signaling mechanism in *B. pertussis* and its effect on the downstream differential gene regulation. In one of our earlier communications, we have theoretically identified the temperature mediated molecular switch that controls the signaling mechanism [22]. In addition, theoretical analysis have been made to understand the role of positive feedback on target gene regulation [22, 23]. A preliminary level of sensitivity analysis suggests that the rate constants associated with the kinase and phosphatase activities are most sensitive among all the rate parameters [22]. To extend these earlier reports further and to understand the role of individual rate parameters on the model output we undertake a theoretical approach in the present study that incorporates correlation coefficient based sensitivity analysis and stochastic optimization. The modular approach we have adopted in the present work is the following. In the first module, we classify the kinetic rate parameters associated with the model according to their sensitivity. We identify the set of sensitive model parameters as a function of the input signal. In the next module, we invoke stochastic optimization technique to generate the sharp molecular switch. Finally, the optimized parameter set has been used to reproduce the features of some novel experimental results [24].

## Methods

### The *bvg* operon

**Fig 1:**
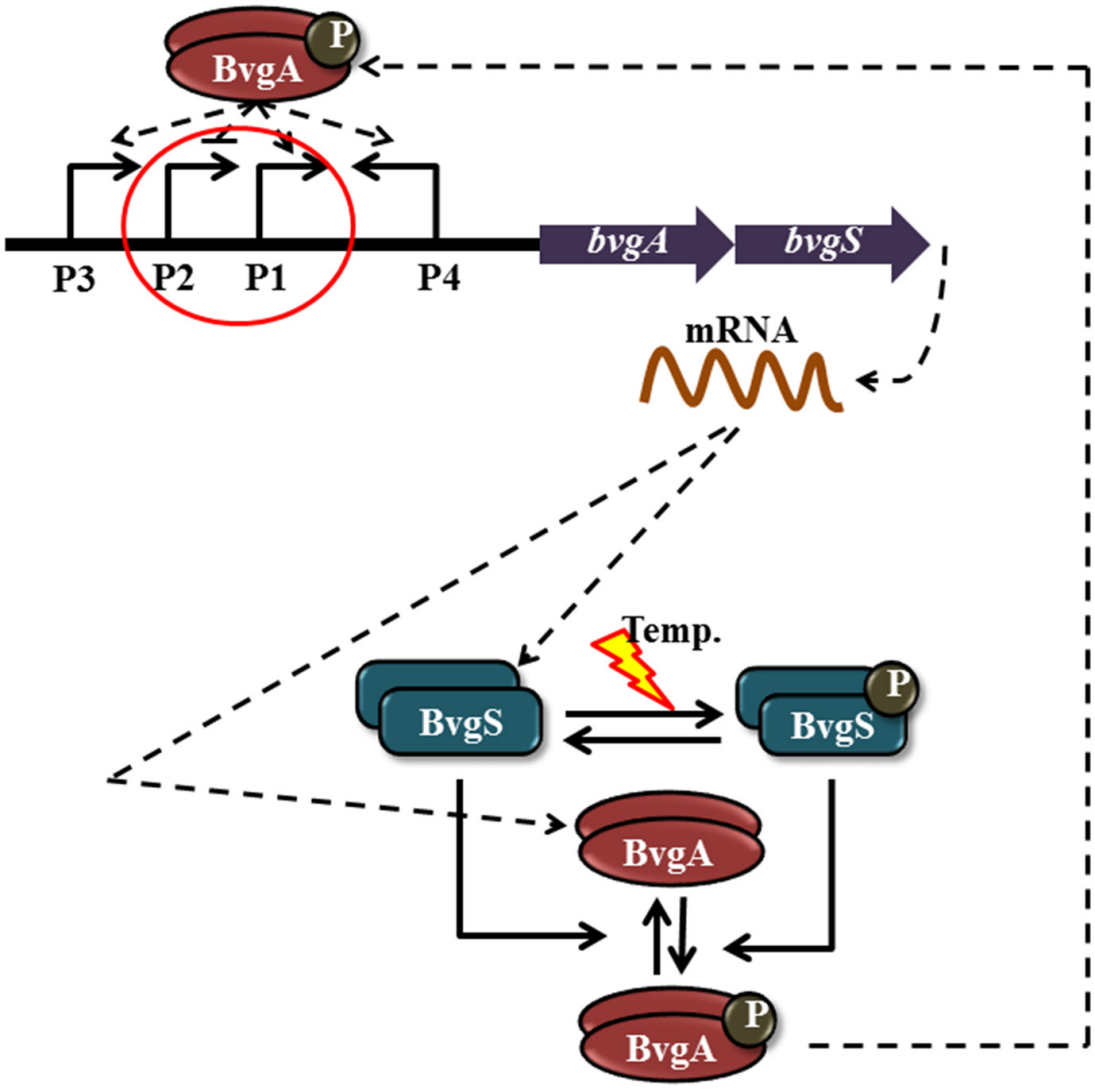
BvgAS signal transduction motif. The signal transduction motif is composed of the phosphotransfer and autoregulation modules. The temperature acts as inducer of the autophosphorylation of the dimer of the sensor kinase protein BvgS (blue). Similarly, dimerized BvgA represents for response regulator in red. P in black sphere stands for phosphate group. Note that, sensor kinase acts both as source and sink for the phosphate group.

Many of the pathogenic secretions of *B. pertussis* are controlled by phosphorylated BvgA, a member of the BvgAS two-component system and is encoded by the *bvg* operon [7, 8]. In *bvg* operon, four promoters (*P*_1_, *P*_2_, *P*_3_, and *P*_4_) together control the production of BvgS and BvgA [25–27] (see Fig 1). Out of the four promoters, *P*_2_ shows constitutive behavior in absence of any external stimulus. However, as *B. pertussis* experiences temperature rise in the surroundings, activity of *P*_2_ goes down. Under the same condition, the rest of the promoters get activated. Activity of *P*_3_ is very low under induction [27] and has been excluded from our model. Similarly, contribution of *P*_4_ has been excluded from our model as *P*_4_ produces anti-sense RNA whose target has not been yet identified. In this connection, recent work by Hot et al. [28] is worth mentioning, where the authors have identified an anti-sense RNA *bprJ2*, regulated by BvgAS TCS, with unknown functionality.

### The BvgAS two-component system

The phosphorylated dimer of the cytosolic response regulator protein BvgA binds at the promoter site as a transcription factor and catalyzes the promoter activation. Upon activation, RNA polymerase transcribes the polycistronic mRNA *m*, which on translation accumulates the pool of the monomeric cognate pair proteins BvgS and BvgA. BvgS, after dimerization, spans in the transmembrane region to sense the external stimulus. In the present work, we denote the dimers of BvgS and BvgA as *S*_2_ and *A*_2_, respectively. Although the sensor and the response regulator proteins first get expressed as a monomer and then dimerize, we do not consider the dimerization kinetics in our model as only the dimer forms of the proteins are of functional interest here. As the temperature in the surroundings increases, *S*_2_ gets autophosphorylated at the histidine residue to form *S*_2*P*_. *S*_2*P*_ then transfers the phosphate group to the aspertate residue of the cognate *A*_2_ through kinase activity, thus producing *A*_2*P*_. *A*_2*P*_ on the other hand, gets dephosphorylated to *A*_2_ due to the phosphatase activity of *S*_2_. As a result, the bifunctional sensor protein BvgS helps in producing the pool of phosphorylated response regulator BvgA which in turn autoregulates its operon as well as regulates the expression of several downstream genes (see Fig 1). For detailed kinetic scheme and parameter set, we refer to Table 1. These parameter set was used earlier to understand the mechanism of molecular switch in *B. pertussis* [22]. Based on the above information the signal transduction module in BvgAS TCS can thus be divided into two parts: the autoregulation motif and the phosphotransfer motif, typical characteristics of bacterial TCS [11, 22]. These two motifs together generate a sharp molecular switch under the induction of temperature increase in the surroundings [22].

**Table 1:** List of kinetics schemes and the values of rate parameters used in the model.

Description	Reaction	kinetic rate constant
Association of *A*_2*P*_ and *P*_*i*_	*P*_*i*_ + *A*_2*P*_ → *P*_*a*_	*k*_*b*_ = 1.024 × 10^−4^ nM^−1^s^−1^
Dissociation of *A*_2*P*_ from *P*_*i*_	*P*_*a*_ → *P*_*i*_ + *A*_2*P*_	*k*_*u*_ = 1.167 × 10^−3^ s^−1^
Basal transcription from *P*_*i*_	*P*_*i*_ → *P*_*i*_ + *m*	*k*_*tp*0_ = 1.9 × 10^−2^ s^−1^
Activated transcription from *P*_*a*_	*P*_*a*_ → *P*_*a*_ + *m*	*k*_*tp*1_ = 4.083 × 10^−2^ s^−1^
Degradation of *m*	*m* → *ϕ*	*k*_*dm*_ = 1.667 × 10^−3^ s^−1^
Translation of *S*_2_ from *m*	*m* → *m* + *S*_2_	*k*_*ss*_ = 6.667 × 10^−4^ s^−1^
Translation of *A*_2_ from *m*	*m* → *m* + *A*_2_	*k*_*sa*_ = 4.167 × 10^−3^ s^−1^
Autophosphorylation of *S*_2_ at 37° *C*	*S*_2_ → *S*_2*P*_	*k*_*ps*_ = 8.333 × 10^−3^ s^−1^
Autodephosphorylation of *S*_2_	*S*_2*P*_ → *S*_2_	*k*_*dps*_ = 3.333 × 10^−3^ s^−1^
Association of *S*_2*P*_ and *A*_2_	*S*_2*P*_ + *A*_2_ → *S*_2*P*_.*A*_2_	*k*_*tf*_ = 8.532 × 10^−3^ nM^−1^s^−1^
Dissociation of *S*_2*P*_.*A*_2_	*S*_2*P*_.*A*_2_ → *S*_2*P*_ + *A*_2_	*k*_*tb*_ = 1.667 × 10^−3^ s^−1^
Phophotransfer from *S*_2*P*_ to *A*_2_	*S*_2*P*_.*A*_2_ → *S*_2_ + *A*_2*P*_	*k*_*ta*_ = 8.333 × 10^−2^ s^−1^
Association of *S*_2_ and *A*_2*P*_	*S*_2_ + *A*_2*P*_ → *S*_2_.*A*_2*P*_	*k*_*pf*_ = 3.413 × 10^−5^ nM^−1^s^−1^
Dissociation of *S*_2_.*A*_2*P*_	*S*_2_.*A*_2*P*_ → *S*_2_ + *A*_2*P*_	*k*_*pb*_ = 1.333 × 10^−3^ s^−1^
Dephosphorylation of *A*_2*P*_ by *S*_2_	*S*_2_.*A*_2*P*_ → *S*_2_ + *A*_2_	*k*_*pa*_ = 5.0 × 10^−2^ s^−1^
Degradation of *S*_2_	*S*_2_ → *ϕ*	*k*_*dp*_ = 1.667 × 10^−4^ s^−1^
Degradation of *A*_2_	*A*_2_ → *ϕ*	*k*_*dp*_ = 1.667 × 10^−4^ s^−1^
Degradation of *S*_2*P*_	*S*_2*P*_ → *ϕ*	*k*_*dp*_ = 1.667 × 10^−4^ s^−1^
Degradation of *A*_2*P*_	*A*_2*P*_ → *ϕ*	*k*_*dp*_ = 1.667 × 10^−4^ s^−1^
Association of *A*_2*P*_ and *P*_*cl*2,*i*_	*P*_*cl*2,*i*_ + *A*_2*P*_ → *P*_*cl*2,*i*1_	*k*_*b*,21_ = 5.119 × 10^−4^ nM^−1^s^−1^
Dissociation of *A*_2*P*_ from *P*_*cl*2,*i*1_	*P*_*cl*2,*i*1_ → *P*_*cl*2,*i*_ + *A*_2*P*_	*k*_*u*,21_ = 1.667 × 10^−4^ s^−1^
Association of *A*_2*P*_ and *P*_*cl*2,*i*1_	*P*_*cl*2,*i*1_ + *A*_2*P*_ → *P*_*cl*2,*i*2_	*k*_*b*,22_ = 1.36 × 10^−3^ nM^−1^s^−1^
Dissociation of *A*_2*P*_ from *P*_*cl*2,*i*2_	*P*_*cl*2,*i*2_ → *P*_*cl*2,*i*1_ + *A*_2*P*_	*k*_*u*,22_ = 1.667 × 10^−4^ s^−1^
Association of *A*_2*P*_ and *P*_*cl*2,*i*2_	*P*_*cl*2,*i*2_ + *A*_2*P*_ → *P*_*cl*2,*a*_	*k*_*b*,23_ = 1.706 × 10^−3^ nM^−1^s^−1^
Dissociation of *A*_2*P*_ from *P*_*cl*2,*a*_	*P*_*cl*2,*a*_ → *P*_*cl*2,*i*2_ + *A*_2*P*_	*k*_*u*,23_ = 1.667 × 10^−4^ s^−1^
Transcription rate from *P*_*cl*2,*a*_	*P*_*cl*2,*a*_ → *P*_*cl*2,*a*_ + *m*_*cl*2_	*k*_*tp*,*cl*2_ = 5.083 × 10^−3^ s^−1^
Association of *A*_2*P*_ and *P*_*cl*3,*i*_	*P*_*cl*3,*i*_ + *A*_2*P*_ → *P*_*cl*3,*i*1_	*k*_*b*,31_ = 8.533 × 10^−5^ nM^−1^s^−1^
Dissociation of *A*_2*P*_ from *P*_*cl*3,*i*1_	*P*_*cl*3,*i*1_ → *P*_*cl*3,*i*_ + *A*_2*P*_	*k*_*u*,31_ = 1.667 × 10^−4^ s^−1^
Association of *A*_2*P*_ and *P*_*cl*3,*i*1_	*P*_*cl*3,*i*1_ + *A*_2*P*_ → *P*_*cl*3,*a*_	*k*_*b*,32_ = 1.365 × 10^−4^ nM^−1^s^−1^
Dissociation of *A*_2*P*_ from *P*_*cl*3,*a*_	*P*_*cl*3,*a*_ → *P*_*cl*3,*i*1_ + *A*_2*P*_	*k*_*u*,32_ = 1.667 × 10^−4^ s^−1^
Association of *A*_2*P*_ and *P*_*cl*3,*a*_	*P*_*cl*3,*a*_ + *A*_2*P*_ → *P*_*cl*3,*i*2_	*k*_*b*,33_ = 1.706 × 10^−6^ nM^−1^s^−1^
Dissociation of *A*_2*P*_ from *P*_*cl*3,*i*2_	*P*_*cl*3,*i*2_ → *P*_*cl*3,*a*_ + *A*_2*P*_	*k*_*u*,33_ = 2.0 × 10^−4^ s^−1^
Transcription rate from *P*_*cl*3,*a*_	*P*_*cl*3,*a*_ → *P*_*cl*3,*a*_ + *m*_*cl*3_	*k*_*tp*,*cl*3_ = 6.16 × 10^−3^ s^−1^

Experimentally, it has been observed that the ratio of total BvgS (*S*_*T*_) to total BvgA (*A*_*T*_) is ≈6 [27] which can be utilized to employ quasi-steady state approximation in analyzing the behavior of the key components of the signal transduction motif at steady state [11, 22]. Considering this, we define

$$\begin{array}{c} S_{T}\approx S_{2}+S_{2P}\;\text{and}\;A_{T}\approx A_{2}+A_{2P}. \end{array}$$ (1)

Furthermore, we define two dimensionless quantities

$$\begin{array}{c} \alpha =\frac{S_{2P}}{S_{T}}\;\text{and}\;\beta =\frac{A_{2P}}{A_{T}}, \end{array}$$ (2)

to analyze our results.

### Sensitivity Analysis

To decipher the sensitivity of the rate parameters on the output of the model we use the tools of correlation coefficient in the present study. The Pearson correlation coefficient (CC) is defined as the covariance between the input and the output parameters with a normalization through division by the product of their standard deviations,

$$\begin{array}{c} r_{k_{i}\beta }=\frac{\sum_{j=1}^{N}\left(k_{ij}-\left\langle k_{i}\right\rangle \right)\left(\beta_{j}-\left\langle \beta \right\rangle \right)}{\sqrt{\sum_{j=1}^{N}{\left(k_{ij}-\left\langle k_{i}\right\rangle \right)}^{2}\sum_{j=1}^{N}{\left(\beta_{j}-\left\langle \beta \right\rangle \right)}^{2}}}, \end{array}$$ (3)

where *r*_*k*_*i*_*β*_ is the CC of the input parameter *k*_*i*_ and output *β*. 〈*k*_*i*_〉 and 〈*β*〉 are the mean (ensemble average) of *k*_*i*_ and *β*, respectively, and *N* is the number of random sampling. If the output rises or falls with the increment of a particular input parameter, it is said to be positively or negatively correlated. The magnitude of the correlation coefficient, which implies the strength of dependency, spans upto ±1 for maximum +ve or -ve association.

The CC can not explain the sensitivity precisely in the case of nonlinear input-output dependencies. For nonlinear but monotonic increasing relation, one can use the Spearman rank correlation coefficient (RCC) which is the calculation of the correlation coefficient after a rank transformation. The partial rank correlation coefficient (PRCC) takes care of the association of an individual input parameter *k*_*i*_ with the output *β* provided that the dependency of all the other *k*_*j*_-s have been eliminated. This makes PRCC the most reliable among all the sampling based sensitivity indices. PRCC can be calculated from the rank correlation matrix (C), where $C_{ij}$ is the RCC between the *i*-th and *j*-th element. The co-factor *P*_*ij*_ of $C_{ij}$ is utilized to calculate PRCC of an input parameter *k*_*i*_ with the output *β* as

$$\begin{array}{c} P_{k_{i}\beta }=-\frac{P_{k_{i}\beta }}{\sqrt{P_{k_{i}k_{i}}P_{\beta \beta }}}. \end{array}$$ (4)

As correlation coefficient measures how much the output of a network is dependent on the particular input parameter, the correlation coefficient is used as an index of sensitivity in this study. The Pearson correlation coefficient (CC), the Spearman rank correlation coefficient (RCC), and the partial rank correlation coefficient (PRCC) have been calculated for a range of signal *k*_*ps*_ and analyzed. For this purpose, we have perturbed each of the input parameters simultaneously and solved the set of coupled rate equations (see S1 Text) to calculate the output *β*. The distribution of the random perturbations are of Gaussian type whose mean is the base value and the variance is ±5% of the base value. In addition, we have used the data set obtained from 10^5^ independent runs to calculate the correlation coefficients.

Sensitivity analysis of any chemical kinetic network suggests an insight about the priority of the cascade inputs. The utility of parameter sensitivity analysis is to classify the input parameters according to their relative impact on the output [29–32]. In the present study, the phosphorylated fraction of the response regulator (*β* = *A*_2*P*_/*A*_*T*_) is taken as the output variable. As the temperature mediated autophosphorylation rate constant *k*_*ps*_ alters, the dynamics of *β* changes. In the present work, we perform sensitivity analysis over all the rate parameters (except *k*_*ps*_) to check the robustness and the relative importance of the parameter set considered for the reaction kinetics involved in the signal transduction.

### Stochastic Optimization

In the present work, we implement simulated annealing (SA) [33, 34] to decipher the correct parameter set to reproduce results of some *in vitro* experiments reported by Jones et al. [24]. At first, we consider the autophosphorylation of the sensor protein (*S*_2_) and phosphotransfer to the response regulator (*A*_2_) as done in the *in vitro* phosphorylation experiment by Jones et al. [24]. Here, it is important to mention that SA simulation is an algorithmic replica of thermodynamical annealing process [33, 34]. In metallurgical annealing, the metal alloy is taken at a very high temperature and then slowly cooled down to get the most thermodynamically stable state. Similarly, in SA, an algorithmic temperature, called the annealing temperature, is defined. The annealing temperature controls the extent of the search space (or the solution space) that is being sampled. During the simulation a randomly chosen variable is allowed to take a move for each sampling. The maximum step length taken in our simulation is of 5 (minimum) −15 (maximum) % with respect to the value of the particular variable at the previous sampling step. To be explicit, for any parameter *k*, the update using SA is done by the rule *k*′ = *k* + *k* × (−1)^*n*^ × *δ* × *r*_*n*_, where *k*′ is the updated value of *k*, *n* is a random integer, *δ* is the amplitude of allowed change (kept between 0.05 and 0.15), and *r*_*n*_ is a random number between 0 and 1. Using these information a cost or objective function is calculated for the new set of variables after each iteration. With the progress of iteration, the cost profile goes down as the output becomes close to the desired value. The cost function at the *i*-th step of the iteration is calculated as

$$\begin{array}{c} {\text{cost}}_{i}=\sum_{j=1}^{M}{\left(\beta_{ex}\left(j\right)-\beta_{T_{at}}\left(j\right)\right)}^{2}, \end{array}$$ (5)

where *β*_*ex*_(*j*) is the experimental value of relative phosphorylation and *β*_*T*_*at*__(*j*) denotes the relative phosphorylation at the algorithmic (annealing) temperature *T*_*at*_ for *j*-th time at the *i*-th step of the simulation. Similarly, to generate the transcript profile we use the following expression

$$\begin{array}{c} {\text{cost}}_{i}=\sum_{j=1}^{M}{\left(m_{ex}\left(j\right)-m_{T_{at}}\left(j\right)\right)}^{2}, \end{array}$$ (6)

with *m*_*ex*_(*j*) and *m*_*T*_*at*__(*j*) being the experimental value of transcript and the simulated value of the same at the algorithmic (annealing) temperature *T*_*at*_, respectively. While going from *i*-th to *i* + 1-th SA step, the cost function may increase or decrease. If the cost function decreases, we accept the move. On the other hand, if it increases we do not discard the move outright. Instead, we subject it to the Metropolis test [35]. If the quantity Δ = cost_*i*_ − cost_*i*−1_ has a positive value, the probability for accepting the move is determined by the function

$$\begin{array}{c} F=\exp(-\frac{\Delta }{T_{at}}). \end{array}$$ (7)

For positive Δ, *F* is always between 0 and 1. For each evaluation of *F*, we invoke a random number *ξ* (say) between 0 and 1. If *F* > *ξ*, we accept the move. Otherwise, the move is rejected. Thus, at very high *T*_*at*_, *F* will be close to 1 and most moves will be accepted, such that a greater region of the search space will be sampled. As the simulation proceeds, *T*_*at*_ is decreased by the *annealing schedule*. Once the correct path towards the global minimum is attained, we need not search the entire space and concentrate on a small region, which will guide us correctly to the global minimum. In other words, as *T*_*at*_ is lowered, a decreasing number of moves pass the Metropolis test. Finally, we recover the correct set of parameter to reproduce the experimental results. In addition, the optimization is carried out until the cost reaches to zero or a low enough steady value and the corresponding data set is taken as optimized set.

The underlying reason to employ stochastic optimization is to optimize the model parameters in a more reliable and efficient manner. The primary target of any optimization method is to minimize a scalar valued objective function or cost function. This makes stochastic optimization technique the most effective method in developing and fabricating a complex system with large number of components. For systems with high nonlinearity, such an approach gets favor over any deterministic method of optimization. This happens due to the implementation of a Markov Chain Monte Carlo search direction in such a way that one can distinguish the global optima from many local optima. To utilize the principles of stochastic optimization, we implement SA technique [33, 34] which has been successfully applied recently to understand the role of different bonding (stacking and hydrogen) interactions on the breathing dynamics of DNA [36, 37].

## Results and Discussion

### Amplification and Switch

In presence of a stimulus, regulatory networks in all living systems need a switching from *off* to *on* state or *vice versa*. Genetic switch, a typical regulatory system, sometimes gets controlled by the output protein through a feedback loop. The presence of a positive feedback motif in the network makes the switching phenomena sharp. As a result, the network response in terms of the population of the network output shows a sharp growth curve even for a small change in the input signal. Typically, amplification in the signal can be classified into two categories, magnitude amplification and sensitivity amplification [38]. Magnitude amplification occurs in ligand-gated ion channels, where ∼10^4^ ions flow as a single ligand molecule binds to the channel protein. Here, the response is much greater than the stimulus. On the other hand, sensitivity amplification is defined as the fractional change in response with respect to a fractional change in the stimulus. The sensitivity amplification can be calculated locally as [38–40]

$$\begin{array}{c} S_{local}=\frac{\Delta response/response}{\Delta signal/signal}, \end{array}$$ (8)

where the change in the input signal (Δ*signal*) is infinitesimally small. The sensitivity amplification can be calculated globally from the signal-response characteristic curve (see Fig 1 of [38]). Following Koshland et al, the order of sensitivity can be classified as ultrasensitive, hyperbolic sensitive and subsensitive [38]. A curve is said to be ultrasensitive if a 10%–90% change in the response can be obtained for a very narrow range (4–5 fold) of the signal [38]. In the case of hyperbolic Michaelian response, the range of the signal is ∼81 [38] and for subsensitive amplification, it is about a few thousand [38].

**Fig 2:**
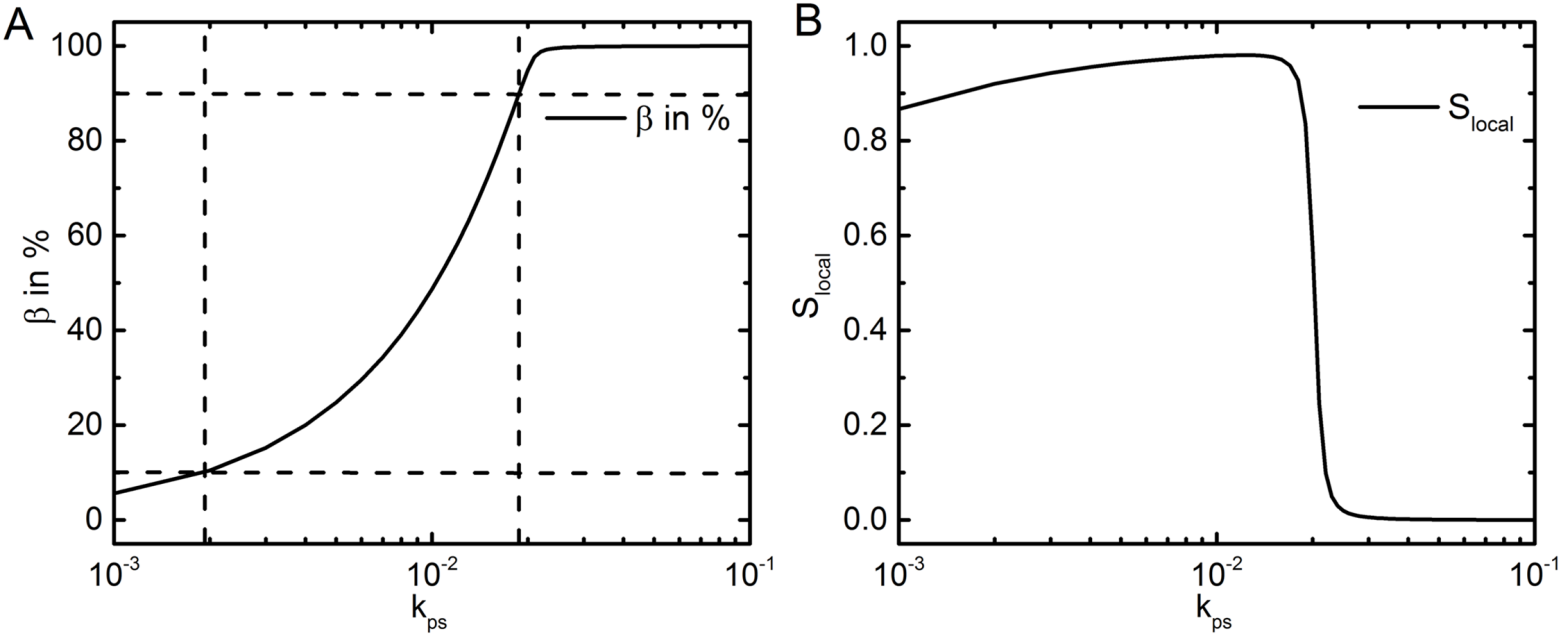
Amplification. **A**. The % amplification and **B**. the sensitivity amplification of the output response *β* with respect to the signal *k*_*ps*_. Note the logarithmic scale in the abscissae.

In the present model, the pool of *A*_2*P*_ is developed when both the phosphotransfer motif and the autoregulation motif are functional. In Fig 2A, we show the amplification of the output response *β*, the fraction of phosphorylated BvgA, as a function of the autophosphorylation rate *k*_*ps*_. The local sensitivity for this model is

$$\begin{array}{c} S_{local}=\frac{\Delta A_{2P}/A_{2P}^{i}}{\Delta k_{ps}/k_{ps}^{i}}. \end{array}$$ (9)

Here, $\Delta A_{2P}=A_{2P}^{f}-A_{2P}^{i}$ and $\Delta k_{ps}=k_{ps}^{f}-k_{ps}^{i}$ with *i* and *f* being the initial and final value, respectively. At lower range of *k*_*ps*_, one can observe a first order ultrasensitive response that reduces to zero order at a high value of the signal. The quantity *S*_*local*_ approaches 1 for small stimulus and sharply falls to 0 for large stimulus (Fig 2B). This happens as the pool of *β* gets saturated at the high value of *k*_*ps*_. If we focus on the global sensitivity with reference to Fig 2A, the amplification of *β* from 10% to 90% occurs in response to a 10 fold increment of the signal. Thus, the sensitivity amplification does not show an ultrasensitive switch. Absence of ultrasensitivity implies that the molecular switch in *B. pertussis* lacks co-operativity in the positive feedback operative at the *bvg* operon.

### Parameter sensitivity analysis

To understand the significance of model parameters on the generation of the molecular switch, we perform sensitivity analysis for all the rate parameters except *k*_*ps*_ (which is treated as the signal) with respect to *β* as the output parameter. The positive feedback network shows a sharp switch with respect to *k*_*ps*_. Thus, sensitivity analysis is performed for a broad range of *k*_*ps*_ values (*k*_*ps*_ = 10^−3^ − 10^−1^) that signify the different regions of the amplification profile shown in Fig 2A. The results thus obtained help us to comment not only on the sensitivity of the rate parameters but also show how the order of sensitivity gets modified with the switch.

CC, RCC and PRCC calculations are carried out as a measure of sensitivity index. The numerical values of the same for three different values of *k*_*ps*_ (10^−3^, 10^−2^ and 10^−1^) are presented in Table 2. At 10^−3^, *β* is very low; at 10^−2^, *β* starts to increase and at 10^−1^, it reaches a high value after a sharp change. The trend of correlation is same in all the three correlation coefficient calculations (CC, RCC, PRCC). In magnitude, CC and RCC are very close representing the linear nature of the output with respect to the input. Since in the calculation of PRCC for a parameter excludes the effect of other variables, the PRCC values are quite higher than that of CC and RCC values.

**Table 2:** CC, RCC, and PRCC values for all the input parameters with output *β* for low, medium and high values of *k*_*ps*_.

Parameters	*k*_*ps*_ = 10^−3^			*k*_*ps*_ = 10^−2^			*k*_*ps*_ = 10^−1^		
	CC	RCC	PRCC	CC	RCC	PRCC	CC	RCC	PRCC
*k*_*b*_	-0.058	-0.055	-0.207	-0.016	-0.017	-0.034	0.003	0.003	0.007
*k*_*u*_	0.054	0.054	0.206	0.007	0.008	0.035	-0.002	-0.003	-0.011
*k*_*tp*0_	-0.075	-0.072	-0.252	-0.006	-0.006	-0.028	0.011	0.010	0.005
*k*_*tp*1_	-0.397	-0.382	-0.814	-0.436	-0.421	-0.844	0.193	0.182	0.569
*k*_*dm*_	0.474	0.456	0.858	0.446	0.433	0.849	-0.196	-0.187	-0.574
*k*_*ss*_	0.033	0.031	0.086	0.025	0.023	0.081	0.342	0.326	0.774
*k*_*sa*_	-0.494	-0.479	-0.869	-0.468	-0.452	-0.861	-0.139	-0.135	-0.457
*k*_*dps*_	-0.005	-0.004	-0.005	-0.004	-0.005	-0.005	-0.218	-0.208	-0.604
*k*_*tf*_	-0.008	-0.013	-0.006	0.002	0.003	0.002	0.554	0.535	0.891
*k*_*tb*_	-0.008	-0.007	-0.003	0.003	0.003	0.002	-0.015	-0.013	-0.033
*k*_*ta*_	0.011	0.010	0.010	-0.006	-0.005	0.007	0.010	0.009	0.031
*k*_*pf*_	-0.416	-0.399	-0.822	-0.444	-0.426	-0.845	-0.342	-0.332	-0.780
*k*_*pb*_	0.016	0.011	0.041	0.017	0.017	0.041	-0.003	-0.003	0.031
*k*_*pa*_	-0.007	-0.006	-0.036	-0.020	-0.020	-0.051	-0.010	-0.011	-0.031
*k*_*dp*_	0.439	0.425	0.845	0.425	0.415	0.836	-0.551	-0.535	-0.892

At *k*_*ps*_ = 10^−3^, *k*_*tp*1_, *k*_*dm*_, *k*_*sa*_, *k*_*dp*_ and *k*_*pf*_ have considerably high correlation coefficient values, where *k*_*tp*1_, *k*_*sa*_ and *k*_*pf*_ are negatively correlated and the others show positive correlation. All the rate parameters show quite similar trends at *k*_*ps*_ = 10^−2^, but at *k*_*ps*_ = 10^−1^, the order as well as the nature of correlation for some parameters get modified. The sensitive parameter at lower *k*_*ps*_ value remains sensitive, however, the nature of correlation becomes opposite for *k*_*tp*1_, *k*_*dm*_ and *k*_*dp*_. At this *k*_*ps*_ value, *k*_*ss*_, *k*_*dps*_ and *k*_*tf*_ also show high correlation which are practically insensitive at a low value of *k*_*ps*_. The change in the nature of sensitivity before and after the *on* state for all the rate parameters are presented in a tabular form in Table 2. With this gross nature of the sensitivity of the parameters, we analyze the nature as well as the order of sensitivity of the rate parameters in the following.

**Fig 3:**
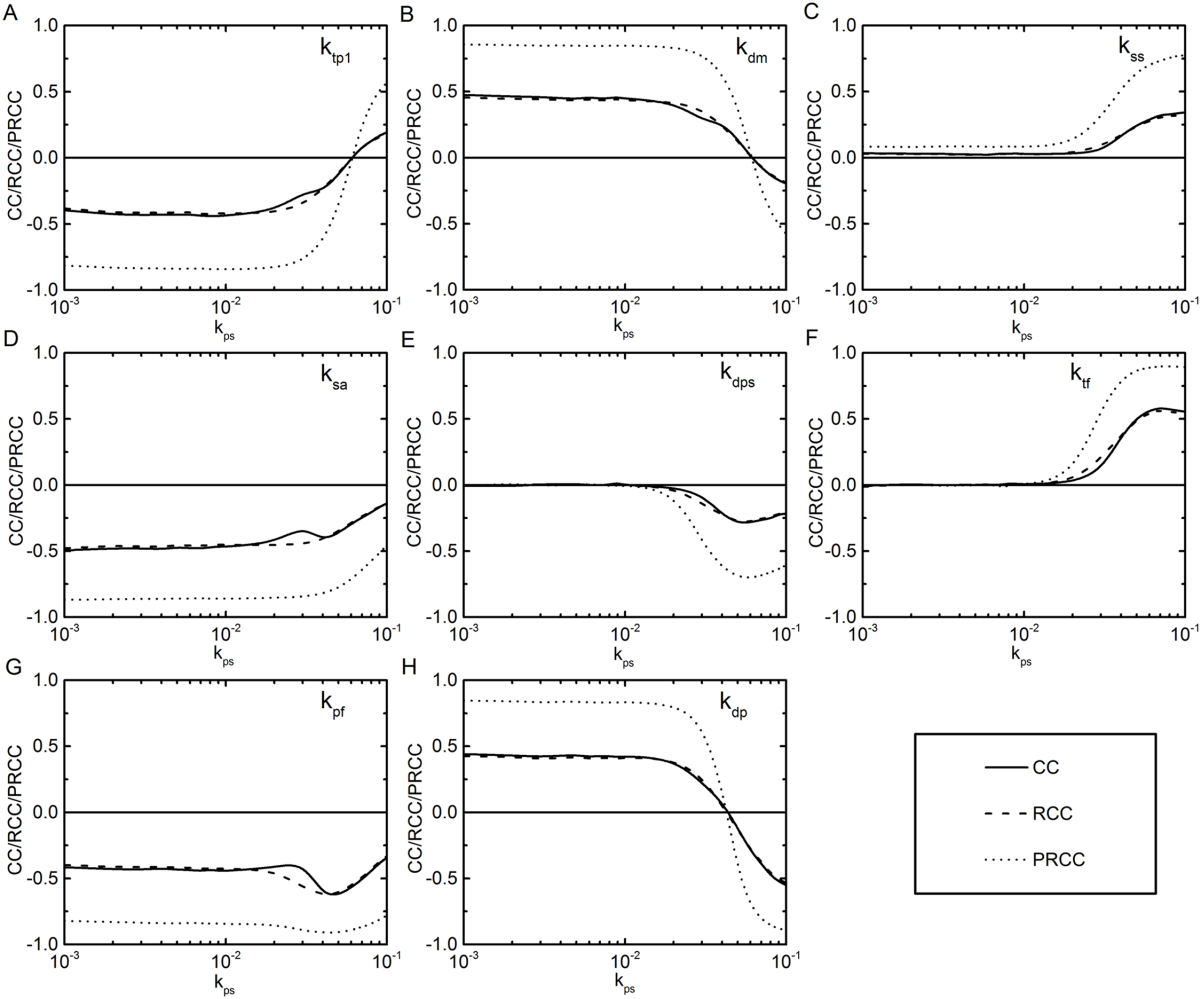
Correlation coefficients. The correlation coefficients CC (solid line), RCC (dashed line) and PRCC (dotted line) as a function of input signal *k*_*ps*_. A, B, C, D, E, F, G and H are for *k*_*tp*1_, *k*_*dm*_, *k*_*ss*_, *k*_*sa*_, *k*_*dps*_, *k*_*tf*_, *k*_*pf*_ and *k*_*dp*_, respectively. Note the logarithmic scale in the abscissae.

The nature of correlation of *k*_*sa*_ does not change much with the change of *k*_*ps*_ value. *k*_*sa*_ is the rate constant for generation of *A*_2_ from mRNA. As the output *β* for the calculation of correlation coefficients is inversely proportional to the *A*_2_ concentration, *k*_*sa*_ shows negative correlation (see Fig 3D). Although it remains negatively correlated, the magnitude of its correlation coefficients (CC, RCC and PRCC) get reduced at a high value of *k*_*ps*_. At high *k*_*ps*_, concentration of *S*_2*P*_ is abundant and phosphotransfer rate increases. Thus, enhanced production of *A*_2_ implies enhanced phosphotransfer to *A*_2_, which in fact, increases the amount of *A*_2*P*_. In a way, if one increases the value of *k*_*sa*_, not only the *A*_2_ concentration increases but a gain in the *A*_2*P*_ concentration also takes place. With these two opposing factors, effectively the order of sensitivity of this parameter reduces at the high value of *k*_*ps*_.

If we consider mRNA generation, the system output *β* varies inversely at low value of *k*_*ps*_. With the increase of mRNA concentration, both the protein concentrations (*S*_2_ and *A*_2_) increase. Increase in *A*_2_ show direct effect on *β*. The rise in *S*_2_ concentration also reduces *β*, as *A*_2*P*_ is dephosphorylated to *A*_2_ by *S*_2_ along with low phosphotransfer (generation of *A*_2*P*_) due to low concentration of *S*_2*P*_. Thus, both the rate constants for mRNA generation (*k*_*tp*0_ and *k*_*tp*1_) show negative correlation at low *k*_*ps*_ value, with *k*_*tp*1_ showing high correlation value than *k*_*tp*0_. In the present work, *k*_*tp*1_ is the rate of mRNA generation from the active state of a promoter (*P*_*a*_) and transformation of *P*_*i*_ → *P*_*a*_ involves loss of *A*_2*P*_. Hence, the effect of *k*_*tp*1_ on the output would be much greater than *k*_*tp*0_. At high *k*_*ps*_, the phosphotransfer motif dominates the whole reaction system thus affecting the mRNA generation. Thus, *k*_*tp*1_ show positive correlation at high *k*_*ps*_ (Fig 3A). The parameter *k*_*dm*_ deals with degradation of mRNA and shows a trend opposite to that of *k*_*tp*1_. Hence, it is positively correlated at low *k*_*ps*_, while showing negative correlation at high *k*_*ps*_ (Fig 3B).

The other two rate parameters that show significant sensitivity after the amplification switch is on are *k*_*ss*_ and *k*_*dps*_. The first one is related to the generation of *S*_2_ from mRNA and the other one is the rate constant for the autodephosphorylation of *S*_2*P*_. At high *k*_*ps*_ value, the phosphotransfer process from *S*_2*P*_ to *A*_2_ becomes significant and eventually the output *β* varies with the concentration of *S*_2*P*_. As with the increase in *k*_*ss*_ and decrease in *k*_*dps*_ amount of *S*_2*P*_ increases, *k*_*ss*_ and *k*_*dps*_ show positive and negative correlation, respectively (Fig 3C and 3E).

At low *k*_*ps*_ value, the kinase reaction does not show any significant effect on the phosphotransfer kinetics as the amount of *S*_2*P*_ is negligible. However, at the high value of *k*_*ps*_, the amount of *S*_2*P*_ increases, thus increasing the phosphorylation of *A*_2_. This leads to the generation of *A*_2*P*_ which, in turn, increases the level of *β*. Hence, it is justified for the rate parameter *k*_*tf*_ to show high positive correlation at the higher value of *k*_*ps*_ (Fig 3F). On the other hand, *k*_*pf*_ is associated with the phosphatase reaction that takes care of the dephosphorylation of *A*_2*P*_; thus showing negative correlation irrespective of the value of *k*_*ps*_ (Fig 3G).

The degradation of all the proteins (*S*_2_, *A*_2_, *S*_2*P*_ and *A*_2*P*_) is controlled by the rate parameter *k*_*dp*_. Hence, it gets correlated with the output (*β*) with a decreasing trend. In the low range of *k*_*ps*_, where the protein pool is minuscule, *k*_*dp*_ shows high positive correlation. However, at the saturation of the protein pool at high *k*_*ps*_ value, the associated correlation of *k*_*dp*_ becomes sharply negative (Fig 3H).

As mentioned earlier, here we have used a Gaussian perturbation of ±5% to calculate the sensitivity of the individual parameters. To check whether the parameter set can withstand any larger perturbation (>±5%) we have systematically increased the magnitude of perturbation by increasing the variance of the Gaussian distribution up to ±20%. The list of resultant data are given in S1 Table for *k*_*ps*_ = 10^−2^ and shows that the CC, RCC and PRCC values remain consistent for ±5%, ±10%, ±15% and ±20% perturbation.

### *In vitro* assay and stochastic optimization: The mutants

The previous subsection describes how efficiently one can decipher the set of reaction rate constants (*k*_*i*_-s) on the basis of their sensitivity towards output *β*. This elucidates the degree of priority of the reactions given in S1 Text. Once the sensitivity of the parameter set is determined, one can target simple motifs present within the complex signaling circuit. One such simple motif is the phosphorylation of BvgA, which has been studied experimentally using some novel mutants [24]. As discussed earlier, activation of the signaling cascade is triggered by the phosphorylation of the transcription factor BvgA by the sensor BvgS. Any alteration through site-directed mutagenesis at the phosphorylation domain of BvgA may influence the phosphorylation kinetics. Two such mutants, T194M and R152H, were employed by Jones et al. in studying the *in vitro* phosphorylation assay [24]. The wild type and the two mutant BvgA were incubated with GST-′BvgS in the presence of [*γ*−^32^P]-ATP and the phosphotransfer kinetics was monitored for 5 min, and the relative amount of phosphorylation was noted at different time points. The relative amount of [*γ*−^32^P]-ATP thus incorporated in BvgA was quantified using phosphoimager. The total protein concentration of BvgS and BvgA used were 0.8 *μ*M and 2.1 *μ*M, respectively. The experimental results suggest that the mutant R152H behaves almost like the WT strain whereas the mutant T194M is heavily impaired in its ability to get phosphorylated. This happens as both arginine (R) and histidine (H) are positively charged and are good acceptor of the negatively charged phosphate group. On the other hand, when the polar threonine (T) residue is replaced by methionine (M) it heavily impairs the phosphorylation capacity.

To examine the performance of the phosphorylated BvgA, one can construct a transcription assay as BvgA on phosphorylation acts as a transcription factor for its promoter and the promoters of the downstream genes. Among the four classes of downstream genes, we opted for *fhaB* (class 2) and *bipA* (class 3) gene as their promoters have high affinity binding sites and show quick response even under low levels of induction [24]. Also, results of *in vitro* transcription assay are available for these classes of genes [24]. High affinity binding site in these two mutants suggests that affinity of phosphorylated transcription factor (BvgA-P) towards the DNA of these two mutants will be high [41]. This information on the other hand suggests that phosphorylated R152H will have higher affinity compared to phosphorylated T194M. Surprisingly, electrophoretic mobility shift assay (EMSA) exhibits reverse result [24], i.e., binding affinity of T194M towards high affinity binding site is higher compared to R152H. One of the plausible mechanism could be the interaction between the negatively charged backbone of the double helix and the positively charged R152H reduces drastically due to histidine (H). Keeping this in mind, we only consider the experimental results of these two genes for optimization purpose.

**Fig 4:**
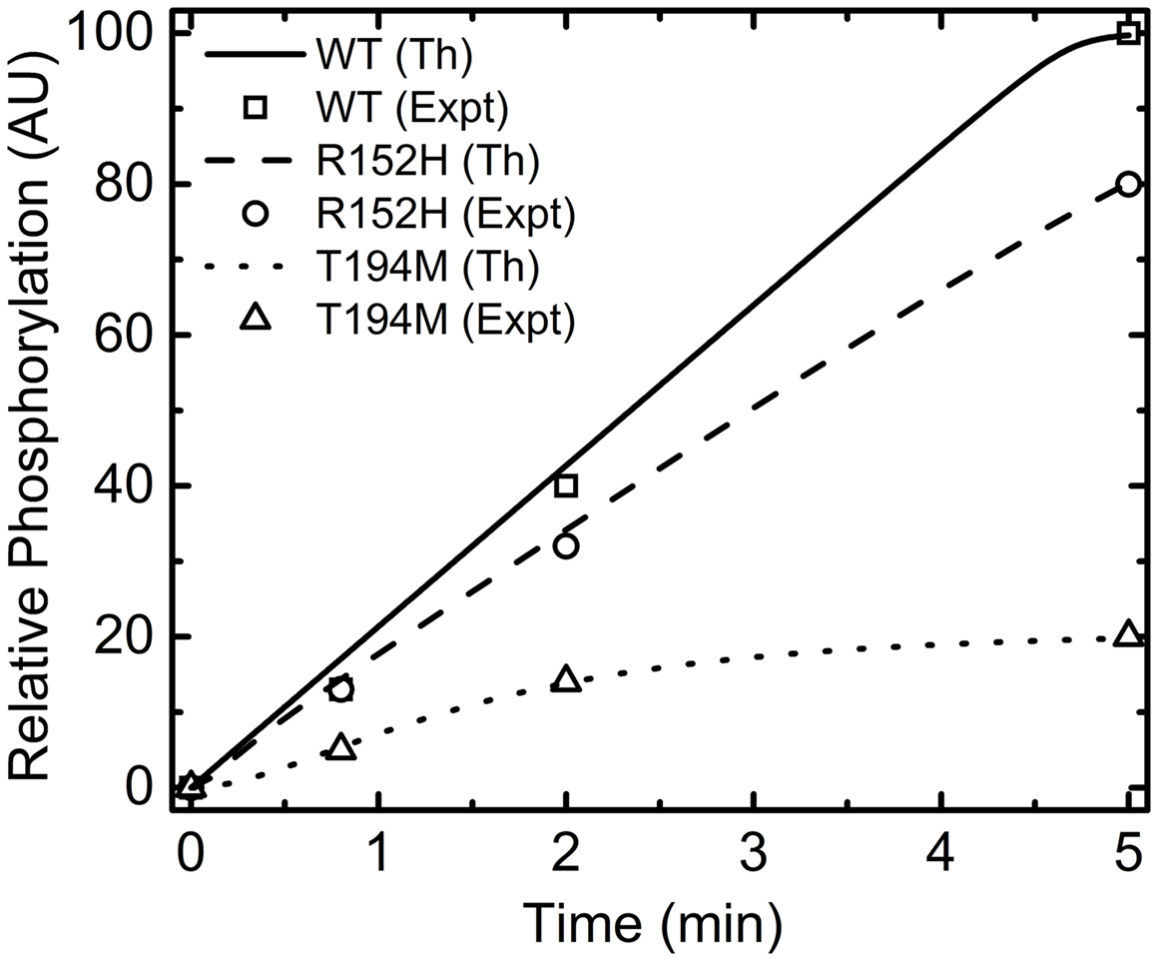
*In vitro* phosphorylation assay. Profiles of *in vitro* phosphorylation assay generated using stochastic optimization. The solid, dashed and dotted lines are for WT, R152H and T194M, respectively. The symbols are experimental results due to Jones el al. [24]. In the figure legend Th and Expt stand for theoretical and experimental data, respectively.

Since we have deciphered the sensitivity of the model parameters, we now focus on reproducing the behavior of some novel mutants mentioned earlier. In the present work, we use simulated annealing, a stochastic optimization technique, to estimate the optimal set of the rate parameters involved in the *in vitro* phosphorylation assay (Table 3). At this point it is important to mention that the process of optimization is computationally expensive as the optimized set of variables is far away from the parameter space from where the sampling is started. The parameter set have to travel a long way to reach the optimized value. Thus, we allowed the parameters to take long step and keep the size up to 15% and carried out 500 independent SA runs. The uncertainty in the output of the SA runs are given in the form of standard deviation in Table 3. The nature of standard deviation suggests that the uncertainty lies within ∼20% of the optimized parameter value. In addition, we have considered only those SA runs where the cost function asymptotically moves towards zero. Otherwise, we do not consider the output of a SA run in our calculation. Keeping this in mind we first generate the profiles of the *in vitro* phosphorylation assay experiment reported by Jones et. al [24]. The kinetic rate constants, optimized using SA, reproduces the experimental profile (the symbols in Fig 4). The associated cost function and the evolution of rate parameters (only 5 out of 500 trajectories) as a function of SA steps are shown in S1 Fig. Fig 4 shows that the phosphorylation ability of R152H is higher than that of T194M. On a relative scale, R152H and T194M could be phosphorylated ∼80% and ∼30%, respectively, compared to the WT strain.

**Table 3:** List of optimized parameters used for the simulation of *in vitro* phosphorylation assay. Here, *x*±*y* stand for the value of optimized parameter *x* with standard deviation *y*. The standard deviation is calculated using the data of 500 independent SA runs.

Parameters	WT	R152H	T194M
*k*_*tf*_	(8.98 ± 2.65) × 10^−3^nM^−1^s^−1^	(4.49 ± 1.20) × 10^−4^nM^−1^s^−1^	(3.50 ± 1.29) × 10^−5^nM^−1^s^−1^
*k*_*tb*_	(1.52 ± 0.44) × 10^−3^s^−1^	(6.68 ± 1.69) × 10^−4^s^−1^	(1.43 ± 0.35) × 10^−5^s^−1^
*k*_*ta*_	(1.77 ± 0.4) × 10^−1^s^−1^	(2.46 ± 0.67) × 10^−2^s^−1^	(1.62 ± 0.36) × 10^−4^s^−1^

**Fig 5:**
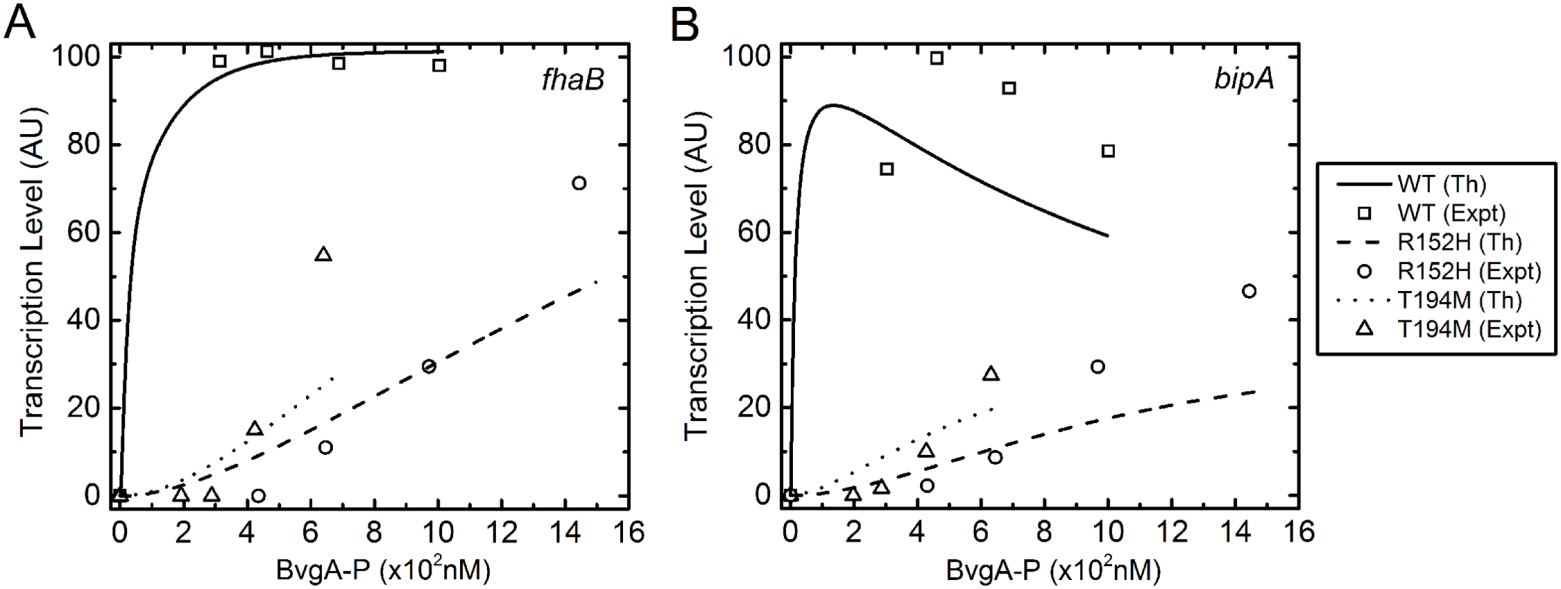
*In vitro* transcription assay. Profiles of *in vitro* transcription assay generated using stochastic optimization for **A**. *fhaB* and **B**. *bipA*. The solid, dashed and dotted lines are for WT, R152H and T194M, respectively. The symbols are experimental results due to Jones el al. [24]. In the figure legend Th and Expt stand for theoretical and experimental data, respectively.

As discussed earlier, on mutation, the successive binding of phosphorylated BvgA (*A*_2*P*_) at different promoters gets affected. The three strains WT, R152H and T194M have been used to observe the effect on DNA-protein interaction through *in vitro* transcription assay. At this point, it is important to mention that the experimental data does not have any error bar that gives an estimation of the uncertainty in the experimental results. The snapshots of the profile of cost function and the parameter optimization with respect to SA steps are shown in S2 and S3 Figs. The optimized parameter set thus obtained could reproduce the qualitative behavior (the solid, dashed and dotted lines in Fig 5) of the *in vitro* experimental results (the open squares, circles and triangles in Fig 5). As mentioned in the previous paragraph, the mutant R152H activates the target genes later compared to the mutant T194M due to its weak interaction with the promoter region of the target gene.

**Table 4:** List of optimized parameters used for the simulation of *in vitro* transcription assay of *fhaB*. Here, *x* ± *y* stand for the value of optimized parameter *x* with standard deviation *y*. The standard deviation is calculated using the data of 500 independent SA runs.

Parameters	WT	R152H	T194M
*k*_*b*,21_	(1.24 ± 0.44) × 10^−5^nM^−1^s^−1^	(1.99 ± 0.64) × 10^−6^nM^−1^s^−1^	(1.53 ± 0.43) × 10^−7^nM^−1^s^−1^
*k*_*u*,21_	(1.51 ± 0.37) × 10^−4^s^−1^	(3.81 ± 0.71) × 10^−6^s^−1^	(1.25 ± 0.33) × 10^−6^s^−1^
*k*_*b*,22_	(1.13 ± 0.39) × 10^−4^nM^−1^s^−1^	(7.96 ± 2.02) × 10^−8^nM^−1^s^−1^	(9.48 ± 2.60) × 10^−6^nM^−1^s^−1^
*k*_*u*,22_	(4.81 ± 1.21) × 10^−5^s^−1^	(4.24 ± 1.38) × 10^−6^s^−1^	(1.02 ± 0.28) × 10^−7^s^−1^
*k*_*b*,23_	(5.96 ± 1.79) × 10^−5^nM^−1^s^−1^	(1.26 ± 0.31) × 10^−6^nM^−1^s^−1^	(3.76 ± 1.2) × 10^−6^nM^−1^s^−1^
*k*_*u*,23_	(5.60 ± 1.80) × 10^−4^s^−1^	(2.06 ± 0.54) × 10^−6^s^−1^	(4.54 ± 1.22) × 10^−6^s^−1^

**Table 5:** List of optimized parameters used for the simulation of *in vitro* transcription assay of *bipA*. Here, *x* ± *y* stand for the value of optimized parameter *x* with standard deviation *y*. The standard deviation is calculated using the data of 500 independent SA runs.

Parameters	WT	R152H	T194M
*k*_*b*,31_	(9.64 ± 2.54) × 10^−5^nM^−1^s^−1^	(3.01 ± 0.84) × 10^−7^nM^−1^s^−1^	(1.62 ± 0.45) × 10^−7^nM^−1^s^−1^
*k*_*u*,31_	(1.07 ± 0.24) × 10^−5^s^−1^	(1.41 ± 0.27) × 10^−5^s^−1^	(4.45 ± 1.02) × 10^−7^s^−1^
*k*_*b*,32_	(5.64 ± 1.38) × 10^−5^nM^−1^s^−1^	(3.07 ± 0.85) × 10^−7^nM^−1^s^−1^	(2.93 ± 0.72) × 10^−6^nM^−1^s^−1^
*k*_*u*,32_	(1.29 ± 0.28) × 10^−4^s^−1^	(1.95 ± 0.52) × 10^−4^s^−1^	(1.95 ± 0.43) × 10^−4^s^−1^
*k*_*b*,33_	(7.21 ± 1.6) × 10^−7^nM^−1^s^−1^	(3.73 ± 1.08) × 10^−7^nM^−1^s^−1^	(9.27 ± 1.98) × 10^−7^nM^−1^s^−1^
*k*_*u*,33_	(3.75 ± 0.62) × 10^−4^s^−1^	(1.76 ± 0.39) × 10^−4^s^−1^	(9.99 ± 2.14) × 10^−4^s^−1^

## Conclusion

The present study undertakes the temperature mediated activation and virulence of BvgAS cascade in the light of sensitivity based optimization. The kinetic model of BvgAS has been simulated for a broad range of sensor protein autophosphorylation that mimics the kinetics of the same quantity due to temperature elevation. The sharp switch in the phosphorylated response regulator is a consequence of positive feedback operative on the *bvgAS* operon. The BvgA-P sharp switch arising due to the integration of the modular structure (the autoregulation and the phosphotransfer module) shows maximum response to the extra-cellular stimulus.

The development of sharp switch has been extensively investigated in the light of sensitivity analysis coupled with stochastic optimization. Our analysis shows the phosphotransfer module to be the more sensitive compared to the autoregulation module. The parameter sensitivity opens up the avenue to classify and explain the role of model parameters in accord with their influence on the steady state dynamics. Once classified, it is possible to tune the most sensitive parameters to regenerate the experimental profiles computationally. The simulated annealing based stochastic optimization performed on three different strains of BvgA (WT, R152H and T194M) could successfully reproduce the characteristics of *in vitro* experimental results. In addition, it helps in the understanding of different nature of attenuation or delay during activation of the mutants. Quantification of such delay is biologically important as a delay in the switch due to temperature elevation brings in obstruction in the virulence to be triggered within the host. The obstruction might become operative in the level of protein-protein interaction or the protein-DNA interaction. Although, *in vivo* fabrication of such synthetic network is difficult, it may be a good starting point in understanding the functionality of stimulus mediated *in vivo* systems. Future experiments leading to target characterization and quantitative measurements of such interactions will help one to build more efficient models.

## Supporting Information

S1 Text General supplementary information. The text contains the detailed kinetic mechanism of BvgAS two-component system, the kinetics of *in vitro* phosphorylation assay and the kinetics of *in vitro* transcription assay.
(PDF)

S1 Fig Optimization of kinetic parameters associated with *in vitro* phosphorylation assay. The cost function and the optimization profiles of the kinetic parameters associated with the simulation of *in vitro* phosphorylation assay results (Fig 4 and Table 3) as a function of SA steps. The colored (red, green, blue, cyan and magenta) lines are representatives of five different SA runs. The black horizontal line represents the base parameter value given in Table 1. Note the logarithmic scale in the ordinates.
(TIF)

S2 Fig Optimization of kinetic parameters associated with *in vitro* transcription assay of *fhaB*. The cost function and the optimization profiles of the kinetic parameters (Table 4) associated with the simulation of *in vitro* transcription assay results of *fhaB* (Fig 5A) as a function of SA steps. The colored (red, green, blue, cyan and magenta) lines are representatives of five different SA runs. The black horizontal line represents the base parameter value given in Table 1. Note the logarithmic scale in the ordinates.
(TIF)

S3 Fig Optimization of kinetic parameters associated with *in vitro* transcription assay of *bipA*. The cost function and the optimization profiles of the kinetic parameters (Table 5) associated with the simulation of *in vitro* transcription assay results of *bipA* (Fig 5B) as a function of SA steps. The colored (red, green, blue, cyan and magenta) lines are representatives of five different SA runs. The black horizontal line represents the base parameter value given in Table 1. Note the logarithmic scale in the ordinates.
(TIF)

S1 Table CC, RCC, and PRCC values for all the input parameters with output *β* for *k*_*ps*_ = 10^−2^ at different range of perturbation. (PDF)
