# Supplementary material for: Deciphering Parameter Sensitivity in the BvgAS Signal Transduction
Source: PLoS One. 2016 Jan 26;11(1):e0147281. doi: 10.1371/journal.pone.0147281 (PMC4727886; doi:10.1371/journal.pone.0147281)
Supplement: S1 Text — The text contains the detailed kinetic mechanism of BvgAS two-component system, the kinetics of in vitro phosphorylation assay and the kinetics of in vitro transcription assay. (PDF) [file pone.0147281.s001.pdf]

# S1 Text

Tarunendu Mapder[1], Srijeeta Talukder[2], Sudip Chattopadhyay[1] Suman K Banik[3]
**1 Department of Chemistry, Indian Institute of Engineering Science and Technology, Shibpur, Howrah 711103, India.**
**2 Department of Chemistry, University of Calcutta, 92 A P C Road, Kolkata 700 009, India.**
**3 Department of Chemistry, Bose Institute, 93/1 A P C Road, Kolkata 700 009, India.**

## The *bvg* operon

In the present model, we have the inducible promoter $P_1$ along with the constitutive promoter $P_2$. For simplicity, we only consider the dynamics of $P_1$ as it is inducible under the increase of temperature. In addition, the inactive and the active states of the promoter are denoted as $P_i$ and $P_a$, respectively, together with the conservation relation $P_i + P_a = 1$. From both the states of the promoter mRNA ($m$), synthesis takes place.

$$\frac{d[P_i]}{dt} = -k_b[A_{2P}][P_i] + k_u[P_a], \quad (1)$$

$$\frac{d[P_a]}{dt} = k_b[A_{2P}][P_i] - k_u[P_a], \quad (2)$$

$$\frac{d[m]}{dt} = k_{tp0}[P_i] + k_{tp1}[P_a] - k_{dm}[m]. \quad (3)$$

## The phosphotransfer kinetics

The activation of BvgS through phosphorylation initiates the phosphotransfer motif. In the course of the addition and removal of the phosphate group to BvgA by BvgS (kinase and phosphatase activity, respectively), two intermediates are generated. The dynamical equations for the intermediates, $S_{2P} \cdot A_2$ and $S_2 \cdot A_{2P}$ are

$$\frac{d[S_{2P} \cdot A_2]}{dt} = k_{tf}[S_{2P}][A_2] - (k_{tb} + k_{ta})[S_{2P} \cdot A_2], \quad (4)$$

$$\frac{d[S_2 \cdot A_{2P}]}{dt} = k_{pf}[S_2][A_{2P}] - (k_{pb} + k_{pa})[S_2 \cdot A_{2P}]. \quad (5)$$

## The protein kinetics

The kinetics associated with the two proteins, BvgS and BvgA and their phosphorylated species due to the autoregulation and the phosphotransfer module can be written in terms of differential equation,

$$\frac{d[S_2]}{dt} = k_{ss}[m] - k_{ps}[S_2] + k_{dps}[S_{2P}] + k_{ta}[S_{2P} \cdot A_2] - k_{pf}[S_2][A_{2P}] + k_{pb}[S_2 \cdot A_{2P}] + k_{pa}[S_2 \cdot A_{2P}] - k_{dp}[S_2], \quad (6)$$

$$\frac{d[A_2]}{dt} = k_{sa}[m] - k_{tf}[S_{2P}][A_2] + k_{tb}[S_{2P} \cdot A_2] + k_{pa}[S_2 \cdot A_{2P}] - k_{dp}[A_2], \quad (7)$$

$$\frac{d[S_{2P}]}{dt} = k_{ps}[S_2] - k_{dps}[S_{2P}] - k_{tf}[S_{2P}][A_2] + k_{tb}[S_{2P} \cdot A_2] - k_{dp}[S_{2P}], \quad (8)$$

$$\frac{d[A_{2P}]}{dt} = -k_b[P_i][A_{2P}] + k_u[P_a] + k_{ta}[S_{2P} \cdot A_2] - k_{pf}[S_2][A_{2P}] + k_{pb}[S_2 \cdot A_{2P}] - k_{dp}[A_{2P}]. \quad (9)$$

# *In vitro* phosphorylation

On autophosphorylation, the kinase activity of BvgS gets triggered and the phosphotransfer to BvgA from BvgS-P occurs. In the case of *in vitro* phosphorylation, the phosphorylation of BvgS occurs in presence of phosphodonor like acetyl phosphate. Consequently, the phosphate group gets transferred to BvgA. The kinetic equations for the *in vitro* phosphorylation scheme can be written as

$$\begin{aligned}
\frac{d[S_{2P}]}{dt} &= k_{ps}([S_T]-[S_{2P}])-k_{dps}[S_{2P}] \\
&\quad -\frac{1}{2}\{(k_{ta}-k_{tb})[S_{2P}\cdot A_2]+k_{tf}[S_{2P}]([A_T]-[A_{2P}])\}, \qquad (10)\\
\frac{d[A_{2P}]}{dt} &= \frac{1}{2}\{k_{tf}[S_{2P}]([A_T]-[A_{2P}])+(k_{ta}-k_{tb})[S_{2P}\cdot A_2]\}, \qquad (11)\\
\frac{d[S_{2P}.A_2]}{dt} &= k_{tf}[S_{2P}]([A_T]-[A_{2P}])-(k_{tb}+k_{ta})[S_{2P}\cdot A_2]. \qquad (12)
\end{aligned}$$

where, $S_T$ and $A_T$ are the total pool of the BvgS and BvgA proteins (phosphorylated and unphosphorylated) and $S_{2P}\cdot A_2$ is the phosphotransfer intermediate.

# *In vitro* transcription

The promoters of different classes of downstream genes get activated by the cooperative binding of the transcription factor, BvgA-P. The class 2 and class 3 genes are modeled here as discussed in the main text. The $P_{clx,i}$-s are the inactive states, the $P_{clx,ik}$-s ($k=1,2,..$) are the inactive intermediates and the $P_{clx,a}$-s are the active states of the genes ($x=2,3$). The promoter kinetics for the class 2 genes are

$$\begin{aligned}
\frac{d[P_{cl2,i}]}{dt} &= k_{u,21}[P_{cl2,i1}]-k_{b,21}[P_{cl2,i}][A_{2P}], \qquad (13)\\
\frac{d[P_{cl2,i1}]}{dt} &= k_{b,21}[P_{cl2,i}][A_{2P}]-k_{u,21}[P_{cl2,i1}]-k_{b,22}[P_{cl2,i1}][A_{2P}]+k_{u,22}[P_{cl2,i2}], \qquad (14)\\
\frac{d[P_{cl2,i2}]}{dt} &= k_{b,22}[P_{cl2,i1}][A_{2P}]-k_{u,22}[P_{cl2,i2}]-k_{b,23}[P_{cl2,i2}][A_{2P}]+k_{u,23}[P_{cl2,a}], \qquad (15)\\
\frac{d[P_{cl2,a}]}{dt} &= k_{b,23}[P_{cl2,i2}][A_{2P}]-k_{u,23}[P_{cl2,a}]. \qquad (16)
\end{aligned}$$

and for the class 3 genes are

$$\begin{aligned}
\frac{d[P_{cl3,i}]}{dt} &= k_{u,31}[P_{cl3,i1}]-k_{b,31}[P_{cl3,i}][A_{2P}], \qquad (17)\\
\frac{d[P_{cl3,i1}]}{dt} &= k_{b,31}[P_{cl3,i}][A_{2P}]-k_{u,31}[P_{cl3,i1}]-k_{b,32}[P_{cl3,i1}][A_{2P}]+k_{u,32}[P_{cl3,a}], \qquad (18)\\
\frac{d[P_{cl3,a}]}{dt} &= k_{b,32}[P_{cl3,i1}][A_{2P}]-k_{u,32}[P_{cl3,a}]-k_{b,33}[P_{cl3,a}][A_{2P}]+k_{u,33}[P_{cl3,i2}], \qquad (19)\\
\frac{d[P_{cl3,i2}]}{dt} &= k_{b,33}[P_{cl3,a}][A_{2P}]-k_{u,33}[P_{cl3,i2}]. \qquad (20)
\end{aligned}$$

The transcript production from the two classes of the activated downstream promoter can be formulated as

$$\frac{d[m_{clx}]}{dt} = k_{tp,clx}[P_{clx,a}]-k_{dm}[m_{clx}]. \qquad (21)$$

where, $P_{clx,a}$ and $m_{clx}$ are the active promoter and produced mRNA of the class of gene ($x=2,3$) and $k_{tp,clx}$ is the synthesis rate constant of the corresponding transcript.
