# Supplementary figures and images for: Deciphering Parameter Sensitivity in the BvgAS Signal Transduction
Source: PLoS One. 2016 Jan 26;11(1):e0147281. doi: 10.1371/journal.pone.0147281 (PMC4727886; doi:10.1371/journal.pone.0147281)

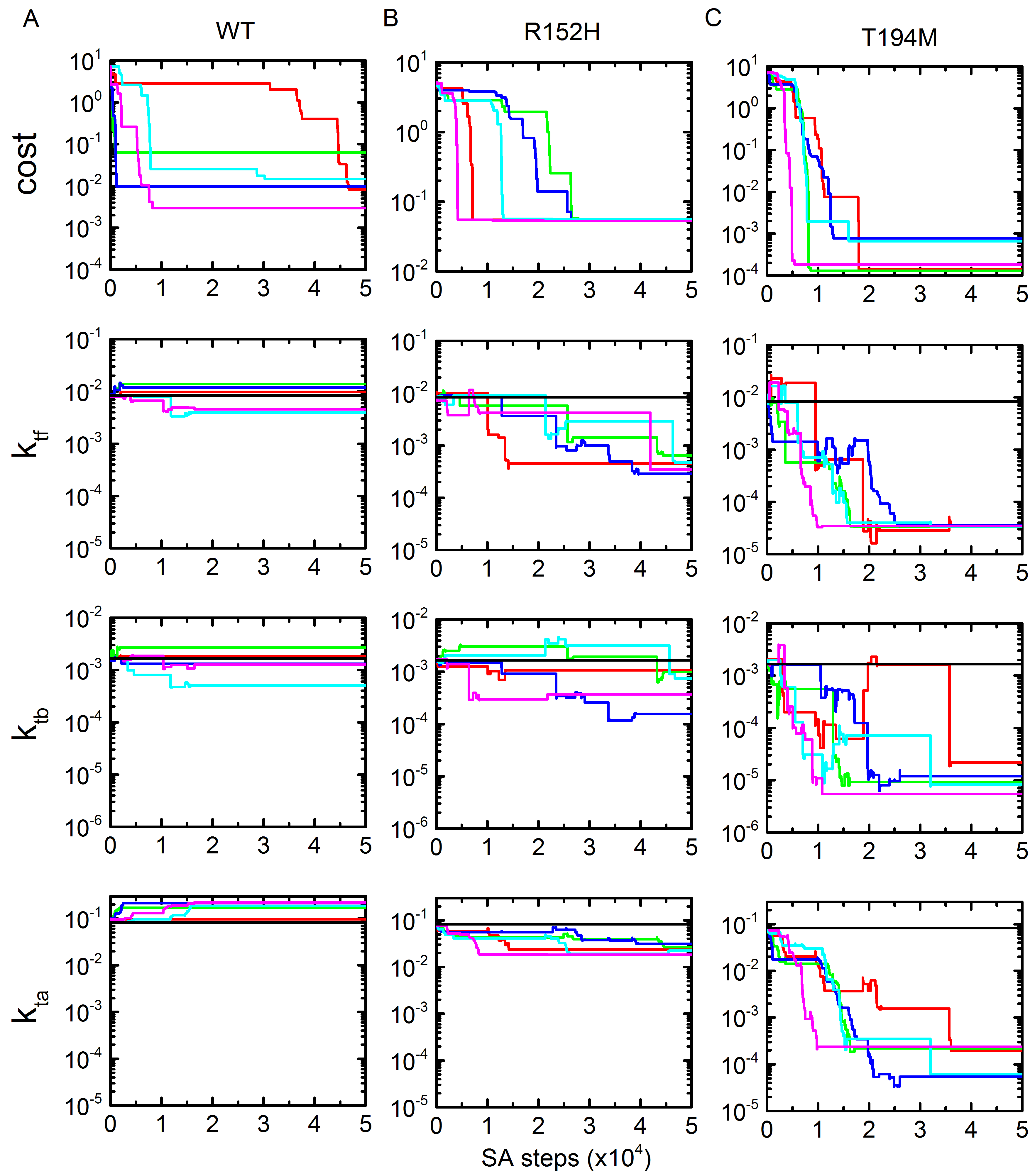

Supplement: S1 Fig — The cost function and the optimization profiles of the kinetic parameters associated with the simulation of in vitro phosphorylation assay results (Fig 4 and Table 3) as a function of SA steps. The colored (red, green, blue, cyan and magenta) lines are representatives of five different SA runs. The black horizontal line represents the base parameter value given in Table 1. Note the logarithmic scale in the ordinates. (TIF) [file pone.0147281.s002.tif]

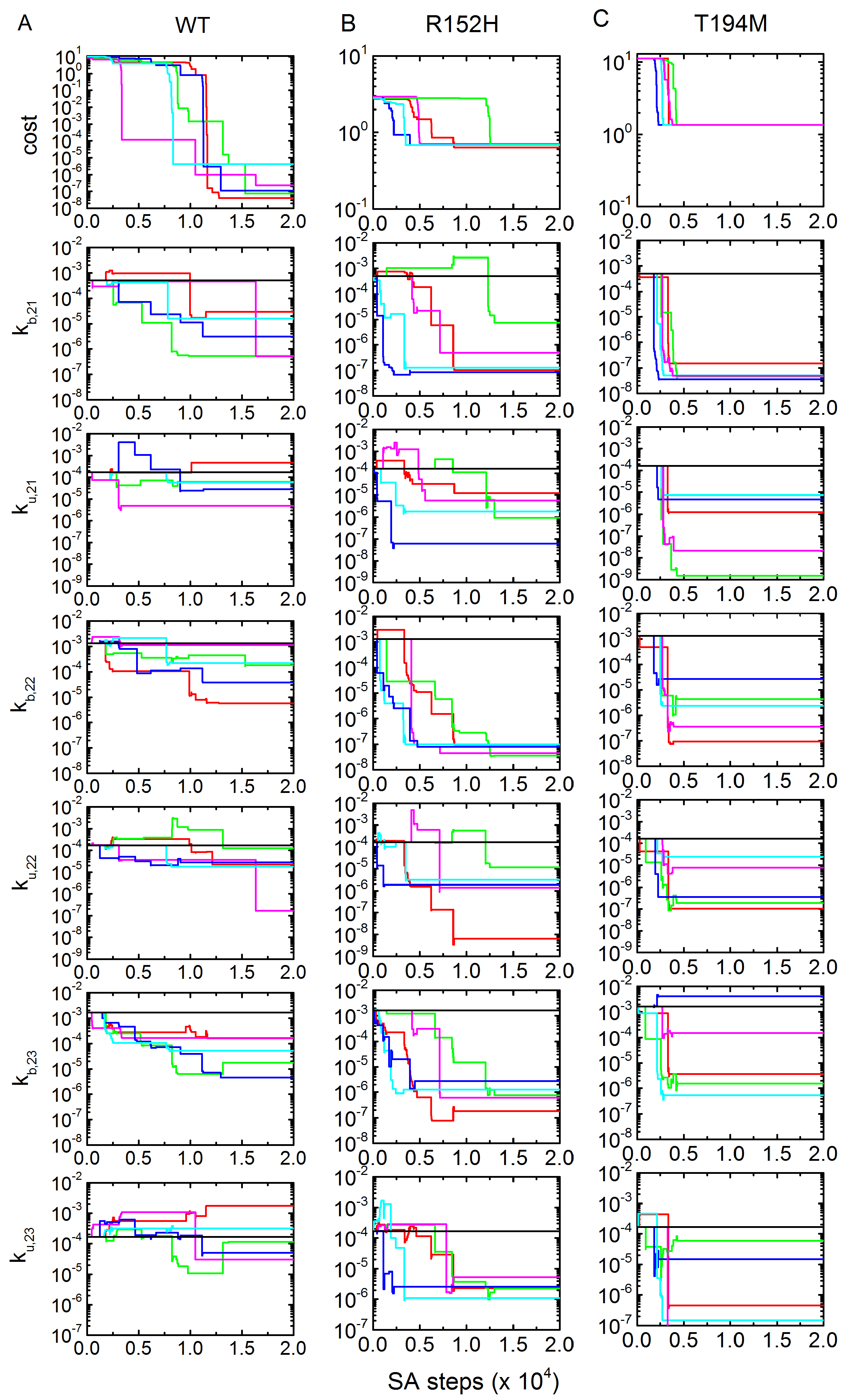

Supplement: S2 Fig — The cost function and the optimization profiles of the kinetic parameters (Table 4) associated with the simulation of in vitro transcription assay results of fhaB (Fig 5A) as a function of SA steps. The colored (red, green, blue, cyan and magenta) lines are representatives of five different SA runs. The black horizontal line represents the base parameter value given in Table 1. Note the logarithmic scale in the ordinates. (TIF) [file pone.0147281.s003.tif]

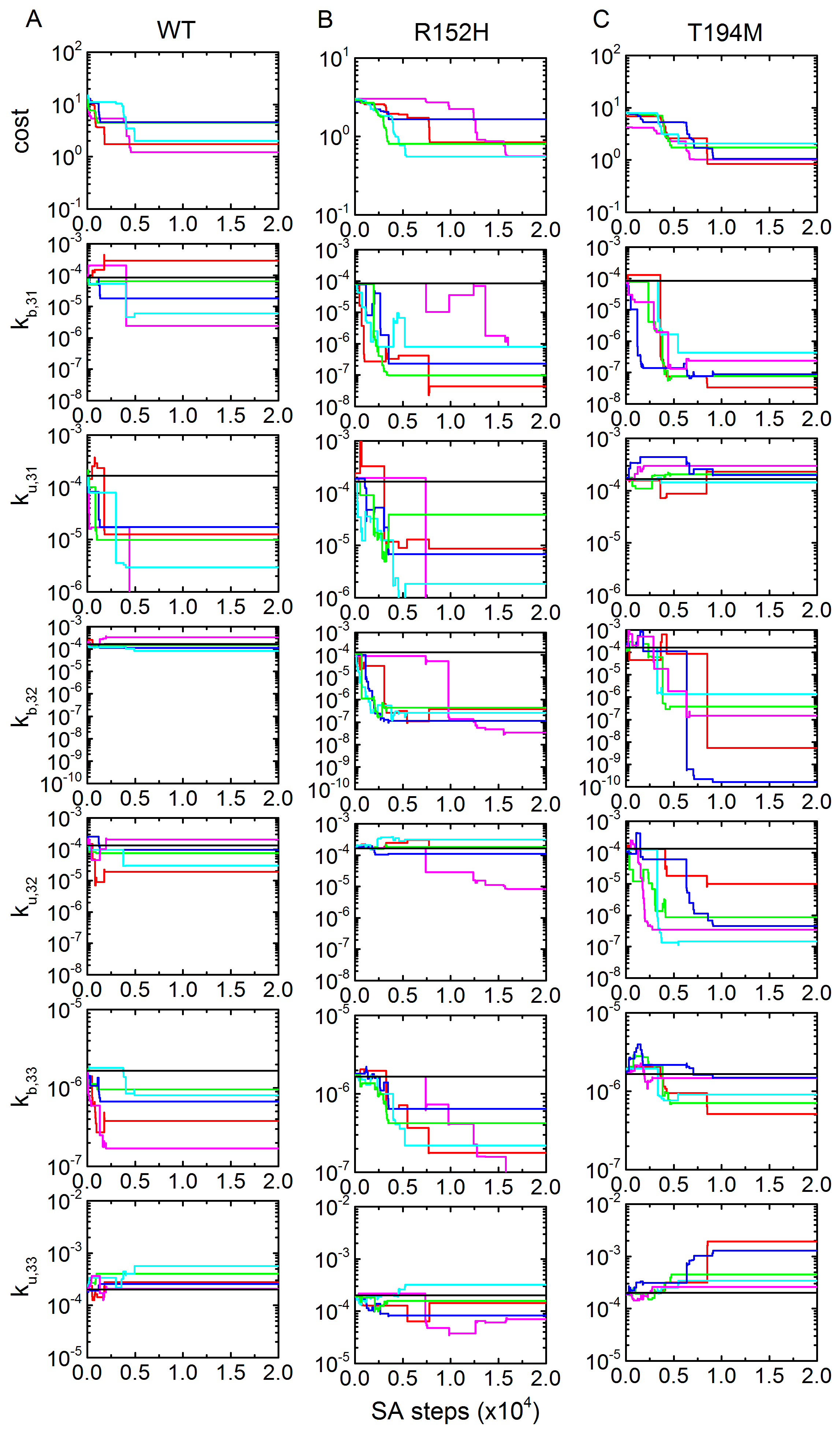

Supplement: S3 Fig — The cost function and the optimization profiles of the kinetic parameters (Table 5) associated with the simulation of in vitro transcription assay results of bipA (Fig 5B) as a function of SA steps. The colored (red, green, blue, cyan and magenta) lines are representatives of five different SA runs. The black horizontal line represents the base parameter value given in Table 1. Note the logarithmic scale in the ordinates. (TIF) [file pone.0147281.s004.tif]
