# Supplementary material for: Deciphering Parameter Sensitivity in the BvgAS Signal Transduction
Source: PLoS One. 2016 Jan 26;11(1):e0147281. doi: 10.1371/journal.pone.0147281 (PMC4727886; doi:10.1371/journal.pone.0147281)
Supplement: S1 Table — (PDF) [file pone.0147281.s005.pdf]

S1 Table. CC, RCC, and PRCC values for all the input parameters with output β for $k_{ps} = 10^{-2}$ at different range of perturbation.

$k_i s$	±5%			±10%			±15%			±20%		
	CC	RCC	PRCC	CC	RCC	PRCC	CC	RCC	PRCC	CC	RCC	PRCC
k_b	-0.016	-0.017	-0.034	-0.016	-0.017	-0.034	-0.015	-0.017	-0.031	-0.013	-0.016	-0.033
k_u	0.007	0.008	0.035	0.007	0.008	0.035	0.007	0.009	0.033	0.008	0.010	0.037
k_{tp0}	-0.006	-0.006	-0.028	-0.006	-0.006	-0.028	-0.006	-0.005	-0.026	-0.007	-0.007	-0.027
k_{tp1}	-0.436	-0.421	-0.844	-0.437	-0.421	-0.844	-0.434	-0.419	-0.841	-0.436	-0.419	-0.842
k_{dm}	0.446	0.433	0.849	0.446	0.433	0.849	0.446	0.431	0.848	0.446	0.431	0.852
k_{ss}	0.025	0.023	0.081	0.025	0.023	0.081	0.025	0.023	0.081	0.024	0.027	0.082
k_{sa}	-0.468	-0.452	-0.861	-0.466	-0.452	-0.861	-0.468	-0.453	-0.861	-0.471	-0.454	-0.857
k_{dps}	-0.004	-0.005	-0.005	-0.004	-0.005	-0.005	-0.004	-0.004	-0.005	-0.004	-0.005	-0.005
k_{tf}	0.002	0.003	0.002	0.002	0.003	0.002	0.002	0.003	0.001	0.002	0.003	0.002
k_{tb}	0.003	0.003	0.002	0.003	0.003	0.002	0.003	0.004	0.002	0.003	0.003	0.002
k_{ta}	-0.006	-0.005	0.007	-0.006	-0.005	0.007	-0.006	-0.005	0.007	-0.006	-0.006	0.007
k_{pf}	-0.444	-0.426	-0.845	-0.444	-0.426	-0.845	-0.438	-0.426	-0.842	-0.443	-0.428	-0.845
k_{pb}	0.017	0.017	0.041	0.014	0.017	0.041	0.017	0.018	0.043	0.013	0.017	0.040
k_{pa}	-0.020	-0.020	-0.051	-0.020	-0.020	-0.051	-0.020	-0.020	-0.048	-0.022	-0.018	-0.047
k_{dp}	0.425	0.415	0.836	0.425	0.415	0.836	0.424	0.414	0.832	0.421	0.417	0.834
